# A one-year hospital system review of plasma next-generation sequencing in a mixed population

**DOI:** 10.1017/ash.2024.460

**Published:** 2024-11-19

**Authors:** Ty C. Drake, Jessica Babic, Audrey Wanger, Violeta Chavez

**Affiliations:** 1 Memorial Hermann –Texas Medical Center, Houston, TX, USA; 2 Department of Pathology and Laboratory Medicine, The University of Texas Health Science Center at Houston, Houston, TX, USA

## Abstract

**Objective:**

To describe the use of plasma next-generation sequencing (NGS) and determine if it provided additional information from routine tests or lead to change in antimicrobial management.

**Design and Setting:**

This retrospective cohort study evaluated patients with a NGS test performed who were admitted to a hospital health system in the greater Houston area between May 2022 and May 2023.

**Patients:**

In total, 143 NGS tests were ordered in the span of one year for 135 unique patients. Most patients were ≥ 18 years (74.1%), White/Caucasian (43.7%), male (61.5%), and immunocompetent (54.1%). Eight patients had repeat tests during the study period, four being after an initial rejected test, and the rest being greater than 7 days from the initial test.

**Results:**

Of the 139 NGS tests performed, 56 (40%) were positive. When compared to routine testing, 49 (35%) were negative when routine testing was negative, 15 (11%) positive were concordant with routine testing, 29 (21%) positive were discordant from routine testing, 17 (12%) negative while routine testing was positive, and 29 (21%) were positive while routine testing was negative. Documented changes in antimicrobial due to NGS occurred after 16 (13.6%) tests, with the majority of these changes occurring in immunocompromised patients (14/16 [88%]).

**Conclusions:**

NGS provided additional data when compared to routine testing but rarely resulted in antimicrobial changes. The majority of changes occurred in immunocompromised patients. Diagnostic stewardship is a vital component for this type of NGS testing and others in which guidelines do not exist.

## Introduction

Clinical microbiology is a science that continues to be vital in the diagnosis of infectious disease (ID). The interpretation in microbiology depends on the quality and type of specimens submitted for analysis. The IDSA and ASM guideline for utilization of microbiology describes the types of samples and testing that aid physicians in quickly and accurately diagnosing ID in their patient.^1^ Although culture and serology remain the gold standard for most ID diagnoses, molecular testing has made significant strides in respiratory and sexually transmitted infections.^2^ The advent of molecular diagnostics and more recently, plasma microbial cell-free DNA (mcfDNA) next-generation sequencing (NGS) has become a coveted method for rapid results. A recently available test uses mcfDNA NGS from plasma to identify potential pathogenic organisms (The Karius Test®, Karius Inc., Redwood City, CA). This test is marketed as a non-invasive rapid detection test for 1,250 clinically relevant bacteria, viruses, fungi, and eukaryotic parasites and recently has added genetic resistance markers to their panel.^3^

The cost of NGS is high and currently not reimbursed by Centers for Medicare and Medicaid Services (CMS). The Karius Test® is currently designated as a Breakthrough Device by the US Food and Drug Admiration (FDA) for immunocompromised patients with suspected lung infections. Karius utilizes proprietary algorithm used to provide the number of organisms present in the patient sample that is above a certain threshold. Metagenomic sequencing, employed in shotgun diagnostics, faces substantial challenges primarily revolving around high costs and limited clinical significance. Shotgun diagnostics can yield vast amounts of information, but distinguishing between pathogenic and commensal organisms, as well as discerning their roles in disease, presents an ongoing challenge. This lack of specificity hampers the translation of metagenomic sequencing results into actionable clinical insights, limiting its current utility in routine diagnostics where cost-effectiveness and meaningful clinical interpretation are paramount.

The goals of this retrospective analysis is to describe the use of plasma NGS and determine if it provided additional information from routine tests or lead to change in antimicrobial management.

## Methods

This retrospective cohort study was performed at an 11-hospital health system in the greater Houston area in hospitalized patients for whom NGS was ordered between May 2022 and May 2023. Repeat tests on the same patient were included if ordered greater than 7 days from previous test. Tests that did not result due to an inadequate sample were excluded.

NGS was ordered via a downtime form based on institutional criteria, which included (1) ordered by an ID physician and (2) discussed with an ID pharmacist and/or a medical microbiologist prior to sending. NGS testing was limited to patients with negative routine testing for at least 48 hours, in addition to other criteria listed in Supplemental Table 1. Plasma was collected by the hospital staff and sent to the laboratory for testing. The plasma NGS testing used in this study was the Karius Test® (Karius Inc., Redwood City, CA).

Data elements including patient demographics, routine testing, and antibiotic changes were collected via retrospective chart review. Patients were considered immunocompromised if diagnosed as having human immunodeficiency virus (HIV), a solid organ transplant, active malignant tumor, or actively on immunosuppressive therapy. Routine testing included cerebrospinal fluid, blood, respiratory, and wound cultures. Additionally, singular viral PCR testing from the aforementioned sites were included. Biopsies sent for broad range sequencing were also analyzed within a 7-day period. Serologies (eg, EBV, CMV, *Rickettsia*, *Brucella*) were also included as routine studies. Yeast species were only identified if from a sterile site or requested by treating team. Enterococcus species were only identified if from blood or requested by treating team. Results were considered concordant for these organisms if NGS detected any species and routine testing did not provide species identification.

Antimicrobial changes were considered directly attributable to the NGS test if the treating team documented in electronic health record that antimicrobial change was made due to NGS testing result.

This intuitional review board of the University of Texas Health Science Center at Houston and Memorial Hermann Hospital (HSC-MS-0920) approved this study.

## Results

A total of 143 tests were performed in 135 patient encounters during the study period. Four samples were rejected from processing due to receipt later than 4 days and not meeting internal quality control standards. Eight patients had repeat tests during the study period, four being after an initial rejected test, and the rest being greater than 7 days apart from the initial test.

Most patients were adults (74.1%), White/Caucasian (43.7%), and male (61.5%). Furthermore, 45.9% patients were immunocompromised: 28 patients with solid organ transplant, 8 patients with malignant tumor, 7 patients with HIV/AIDS, and 19 with other immunocompromising conditions (Table 1). Despite institutional testing criteria limiting NGS testing to IDs physicians and in patients with negative routine testing for at least 48 hours, there were 11% (5/134) ordered by non-ID providers and 13% (7/134) within 2 days of admission.

**Table 1.** Patient encounter demographics

	Adult n = 100	Pediatric n = 35	Total n = 135
**Age, years, median**	58	4	49
**Male, no. (%)**	66 (66)	17 (49)	83 (62)
**Race, no. (%)**			
White	49 (49)	10 (29)	59 (44)
Black/African American	24 (24)	7 (20)	31 (23)
Hispanic	17 (17)	12 (34)	29 (21)
Asian	4 (4)	1 (3)	5 (4)
Other	6 (6)	5 (14)	11 (8)
**Immunocompromised, no. (%)**	52 (52)	10 (29)	62 (46)
Solid organ transplant	25 (25)	3 (9)	28 (21)
HIV	7 (7)	– (0)	7 (5)
Malignant tumor	7 (7)	1 (3)	8 (6)
Other	13 (13)	6 (17)	19 (14)
**Length of stay, days, median**	19	18	20

Of the 139 tests completed, 73 were positive for at least one organism. Thirty one were positive for more than one organism (Table 2). The diversity of microorganisms identified were the following: 31 gram-positives, 28 gram-negatives, 23 anaerobes, 5 atypical, 14 fungi, and 32 viruses (Supplemental Table 2).

**Table 2.** NGS test characteristics

	Adult tests n = 106	Pediatric tests n = 37	Total tests n = 143
**Total tests, no. (%)**			
Positive	56 (53)	17 (46)	73 (51)
Negative	47 (44)	19 (51)	66 (46)
Rejected	3 (3)	1 (3)	4 (3)
**Repeat tests, no. (%)**	5 (5)	3 (8)	8 (6)
**Time to test from admission, days, median**	7	8	7
**Organisms identified per test, no. (%)**			
0	47 (44)	19 (51)	66 (46)
1	30 (28)	12 (32)	42 (29)
2	15 (14)	5 (14)	20 (14)
3	2 (2)	–	2 (1)
4	3 (3)	–	3 (2)
5	4 (4)	–	4 (3)
6	1 (2)	–	1 (1)
7	1 (1)	–	1 (1)
**Ordering specialty, no. (%)**			
Infectious disease	87 (82)	37 (100)	124 (87)
Heart failure	12 (11)	– (0)	12 (8)
Critical care	3 (3)	– (0)	3 (2)
Unknown	4 (4)	– (0)	4 (3)

In total, NGS identified 92 additional organisms (65 bacterial, 19 viral, and 8 fungal) but did not identify 48 organisms that were confirmed from routine microbiological testing (30 bacterial, 11 viral, 6 fungal, and 1 parasite). NGS was concordant for 39 organisms (22 bacterial, 13 viral, and 6 fungal) when compared to routine testing (Supplemental Table 3). When comparing each test, 49 NGS samples were negative when routine testing was also negative, 15 positive results were concordant with routine testing, 29 positive results were discordant from routine testing, with 11 being completely discordant, 17 were negative while routine testing had a positive result, and 29 were positive while other testing was negative (Table 3). Concordant results are based on all organisms matching when comparing NGS results and routine testing. Each NGS test and corresponding routine testing can reviewed in Supplemental Tables 6–9.

**Table 3.** NGS versus routine tests organism concordance

	Adult tests n = 103	Pediatric tests n = 36	Total tests n = 139
**NGS and routine tests concordant, no. (%)**	11 (11)	4 (8)	15 (10)
NGS and routine tests discordant, no. (%)*	21 (20)	8 (22)	29 (21)
NGS and routine tests completely discordant	7 (33)	4 (50)	11 (38)
**NGS positive and routine tests negative, no. (%)**	24 (23)	5 (17)	29 (22)
**NGS negative and routine tests positive, no. (%)**	10 (10)	7 (19)	17 (12)
**NGS and routine tests both negative, no. (%)**	37 (36)	12 (33)	49 (35)

*Based on at least one mismatched organism. Additional organisms were not included in calculations.

Out of the 139 tests, 52 had antibiotics changed within 2 days of the NGS result being reported. Sixteen of these changes were directly attributed to the NGS result (9 escalations and 7 de-escalations), with the majority of these changes occurring in immunocompromised patients (14/16, 88%) (Table 4).

**Table 4.** Antimicrobial changes

	Adults (n = 103)	Pediatric (n = 36)	Total n = 139
**Tests with antimicrobial changes within 2 days, no (%)**	**39 (38)**	**13 (36)**	**52 (37)**
Escalations	24 (63)	4 (31)	28 (54)
De-escalations	16 (41)	7 (54)	23 (44)
Antimicrobial selection	1 (3)	2 (15)	24 (46)
Antimicrobial change in immunocompromised patients	25 (64)	6 (46)	31 (60)
**Tests with antimicrobial changes due to NGS, no. (%)**	**13 (13)**	**3 (8)**	**16 (12)**
Escalations	8 (62)	1 (33)	9 (56)
De-escalations	5 (39)	2 (67)	7 (44)
Antimicrobial change in immunocompromised patients	1 (8)	2 (67)	14 (88)

## Discussion

Plasma NGS demonstrated limited benefit in our patient population. It is difficult to ascertain which patients would benefit most consistently from NGS, as there was no apparent pattern to the patients who had positive results, nor did the result of NGS change therapy in most cases. However, there does appear to be a lower threshold to change therapy based on NGS in immunocompromised patients. Testing at will with no additional criteria is likely not appropriate and should not replace a thorough history and physical by the clinician.^4^

Thirty-seven percent of tests had antimicrobial changes within 2 days, with only a third of these being documented due to NGS. Although the impact of NGS varies significantly in current literature (7% to 56%^5^), our findings appear to be inline with others’ experiences. Determination of NGS impact varies greatly among studies, mostly being based on clinical judgment. NGS uses a shotgun approach for detecting organisms, making it hard to determine commensal organisms versus pathogenic. It is up to the treating physician to determine if therapy should be changed based off of the NGS results and patient presentation. Recent literature has made an attempt to standardize criteria for clinical impact of NGS^6^ but still relies heavily on clinical judgment.

There was little correlation found between NGS and routine culture concordance or discordance with antimicrobial changes. Fifty percent of antimicrobial changes due to NGS were when we already had positive cultures of these, 3 NGS results were completely discordant with routine testing. These results offer little insight into understanding the number of organisms which appear in the NGS versus regular culture. Presumably, molecular testing is more sensitive; however, with different results in culture and NGS, the question becomes whether the NGS testing is sensitive enough to determine infectious causes. Furthermore, when a possible pathogen is identified it can be hard to direct therapy given no susceptibilities are available. In one case, NGS identified *Pseudomonas aeruginosa* and the patient was originally started on meropenem; however, once the cultures grew, the susceptibilities showed resistance to meropenem. The most recent panel released by NGS will now provide some antimicrobial resistance data, potentially reducing inappropriate therapy in the future for select organisms.

Clouding the picture further, a negative NGS result does not guarantee the absence of infection. In this review, 17 patients (12%) had pathogens on routine testing which were not identified by the NGS (Table 3), suggesting that NGS testing may not be a suitable method to “rule out” infectious causes. This was not isolated to our review. Similarly, Bergin et al. found that the addition of NGS in immunocompromised patients with pneumonia identified a potential pathogen in 21 more patients compared to usual care but failed to identify similar pathogens compared to usual care in 25 patients.^7^ A point to remember is that NGS results may have a number of organisms that will appear in the analytical results, though only the organisms recorded above a proprietary threshold will be reported in the patient result. In cases where the physician is concerned about an organism, a call to the company can lead to a conversation about other organisms present which did not exceed the infection threshold. In addition, the list of organisms that NGS is currently capable of detecting is not exhaustive. One deceased patient had a blood sample taken for NGS testing 2 days prior to death. The autopsy revealed that the immediate cause of death was aortic root rupture secondary to aortic valve replacement with disseminated fungal disease. The Formalin-Fixed Paraffin-Embedded blocks from autopsy were sent for sequencing and revealed that the organism causing the infection was an *Exerohilum* spp., which this NGS currently does not identify.

Although there are short comings to NGS testing, there are instances in which NGS test resulted in an organism that was not and likely would not have been identified or would have resulted in delayed treatment with current available testing. In one case, NGS testing revealed a *Legionella longbeachae* which did cause a change in therapy. This organism is not easily culturable and would not have been identified in other routine studies. In another case, the identification of a *Mycobacterium abscessus* from a pediatric patient with a tooth abscess was detected 4 days prior to AFB gram stain. Torque teno virus, which has no commercial diagnostic test, has popped up on the radar in the clinical laboratory with NGS. It has been associated with liver disease, though it has also been found in healthy individuals.^8^

Although our study attempted to be as objective as possible, by only looking at the antimicrobial changes and discordance between routine tests, there are some limitations to this. First, we are unable to capture any possible potential advantages and disadvantages to using NGS testing. For example, avoidance of and/or unnecessary further diagnostic evaluation prompted by NGS. Second, we only looked at cultures collected within a 7-day window before or after the NGS test. Although this gives us an accurate representation of potential clinical status around the time of NGS, there were likely organisms that were present when NGS was collected that were not identified by routine tests during this time frame. Lastly, this review was limited greatly by chart documentation and ordering of NGS via a downtime form. Documentation surrounding reason for ordering and result utilization are limited, and ordering criteria was loosely followed, creating a more heterogeneous patient population outside of the institutional criteria.

There is no doubt that novel results from case studies are impressive.^9–11^ However, it is difficult to ascertain when NGS testing should be utilized, and it is unrealistic to utilize on all patients when the culprit of infection is unidentifiable by routine methods due to cost. As NGS becomes prominent and referenced as a potential diagnosis option in guidelines, a consensus-driven guidance would be helpful.^12^ However, based on the multiple published multi-center accounts, there does not appear to be a clear-cut population that would benefit from routine NGS testing.^6,13–15^ A continuous diagnostic stewardship program is prudent as the cost of NGS is high and does not often provide conclusive diagnostic results.

## Supporting information

Drake et al. supplementary material Drake et al. supplementary material

## Supplementary material

For supplementary material accompanying this paper visit http://doi.org/10.1017/ash.2024.460. click here to view supplementary material

## References

[1] Miller JM , Binnicker MJ , Campbell S , et al. A guide to utilization of the microbiology laboratory for diagnosis of infectious diseases: 2018 update by the infectious diseases society of America and the American society for microbiology. Clin Infect Dis 2018;67:813–816. doi: 10.1093/cid/ciy584 30169655

[2] Muralidhar S. Molecular methods in the laboratory diagnosis of sexually transmitted infections. Indian J Sex Transm Dis AIDS 2015;36:9–17. doi: 10.4103/0253-7184.156686 26392648 PMC4555911

[3] Karius. Karius test, https://kariusdx.com/. Accessed July 31, 2024.

[4] Davis JLMJ . History and Physical Examination. 2 ed. Murray and Nadel’s Textbook of Respiratory Medicine; 2016. 263–277.

[5] Morales M. The next big thing? Next-generation sequencing of microbial cell-free DNA using the Karius test. Clin Microbiol Newsl 2021;43:69–80.

[6] Hogan CA , Yang S , Garner OB , et al. Clinical impact of metagenomic next-generation sequencing of plasma cell-free DNA for the diagnosis of infectious diseases: a multicenter retrospective cohort study. Clin Infect Dis 2021;72:239–245. doi: 10.1093/cid/ciaa035 31942944

[7] Bergin SP , Chemaly RF , Dadwal SS , et al. Plasma microbial cell-free DNA sequencing in immunocompromised patients with pneumonia: a prospective observational study. Clin Infect Dis 2024;78:775–784. doi: 10.1093/cid/ciad599 37815489 PMC10954333

[8] Webb B , Rakibuzzaman A , Ramamoorthy S. Torque teno viruses in health and disease. Virus Res 2020;285:198013. doi: 10.1016/j.virusres.2020.198013 32404273

[9] Caldararo M , Algazaq J , Schmidt E , et al. Atypical pathogens presenting with pulmonary consolidations detected by cell-free DNA next-generation sequencing in patients with hematologic malignancies. Infect Dis Clin Pract 2022;30(2):e1101. doi: 10.1097/ipc.0000000000001101 PMC9109825 35586753

[10] Downey RD , Russo SM , Hauger SB , et al. Identification of an emergent pathogen, bartonella vinsonii, using next-generation sequencing in a patient with culture-negative endocarditis. J Pediatric Infect Dis Soc 2021;10:213–216. doi: 10.1093/jpids/piaa014 32092135 PMC11838815

[11] Kalyatanda G , Rand K , Lindner MS , et al. Rapid, noninvasive diagnosis of balamuthia mandrillaris encephalitis by a plasma-based next-generation sequencing test. Open Forum Infect Dis 2020;7:ofaa189. doi: 10.1093/ofid/ofaa189 PMC7371414 32715017

[12] Fowler VG , Durack DT , Selton-Suty C , et al. The 2023 duke-international society for cardiovascular infectious diseases criteria for infective endocarditis: updating the modified duke criteria. Clin Infect Dis 2023;77:518–526. doi: 10.1093/cid/ciad271 37138445 PMC10681650

[13] Bell DT. Deciphering the potential of plasma cell-free metagenomic next-generation sequencing using the Karius test. Curr Opin Infect Dis 2023;36:420–425. doi: 10.1097/QCO.0000000000000942 37493238

[14] Francisco DMA , Woc-Colburn L , Carlson TJ , Lasco T , Barrett M , Al Mohajer M. The effect of a plasma next-generation sequencing test on antimicrobial management in immunocompetent and immunocompromised patients-A single-center retrospective study. Antimicrob Steward Healthc Epidemiol 2023;3:e31. doi: 10.1017/ash.2022.356 36865703 PMC9972541

[15] Wilson MR , Sample HA , Zorn KC , et al. Clinical metagenomic sequencing for diagnosis of meningitis and encephalitis. N Engl J Med 2019;380:2327–2340. doi: 10.1056/NEJMoa1803396 31189036 PMC6764751

