# Supplementary material for: A one-year hospital system review of plasma next-generation sequencing in a mixed population
Source: Antimicrob Steward Healthc Epidemiol. 2024 Nov 19;4(1):e211. doi: 10.1017/ash.2024.460 (PMC11574590; doi:10.1017/ash.2024.460)
Supplement: Drake et al. supplementary material [file S2732494X24004601sup001.docx]

**Supplemental Table 1. NGS Ordering Criteria**

|  | **Criteria for ordering NGS** |
| --- | --- |
| **Culture negative endocarditis^a^** | - Blood cultures negative >48 hours - ECHO with valvular vegetation - Serologies sent for Coxiella/Brucella (given relevant exposures |
| **Immunocompromised or critically ill** | - Patient not improving and without obvious source - Pan-culture negative for > 48 hours - Ongoing fever |
| **Fever of unknown origin** | - Patient not improving and without obvious source and despite broad-spectrum antibiotics - Pan-culture negative >48 hours - Full infectious workup negative - Ongoing fever |
| **Community acquired pneumonia^b^** | - BAL cultures with no growth - Continued signs and symptoms of pneumonia or worsening hypoxemia - Patient not improving despite broad-spectrum antibiotics |
| ^a^If valve tissue available, consider sending for 16s/28s targeted sequencing  ^b^Currently no clinical data in adults | |

**Supplemental Table 2. Organism Distribution**

|  | Adult  (n=111) | Pediatric  (n=22) | Total  (n=133) |
| --- | --- | --- | --- |
| Gram Negative, No.  *Pseudomonas aeruginosa*  *Escherichia coli*  *Stenotrophomonas maltophilia*  *Pseudomonas pseudoalcaligenes*  *Neisseria gonorrhoeae*  *Acinetobacter haemolyticus*  *Serratia ureilytica*  *Cardiobacterium valvarum*  *Neisseria sicca*  *Capnocytophaga sputigena*  *Enterobacter cloacae complex*  *Morococcus cerebrosus*  *Klebsiella aerogenes*  *Klebsiella quasipneumoniae*  *Achromobacter ruhlandii* | 20  5  4  1  1  1  1  1  ─  1  1  1  1  1  1  ─ | 8  4  1  1  ─  ─  ─  ─  1  ─  ─  ─  ─  ─  ─  1 | 28  9  5  2  1  1  1  1  1  1  1  1  1  1  1  1 |
| Gram Positive, No.  *Enterococcus faecium*  *Staphylococcus epidermidis*  *Enterococcus faecalis*  *Rothia mucilaginosa*  *Streptococcus pneumoniae*  *Enterococcus raffinosus*  *Rothia dentocariosa*  *staphylococcus aureus*  *Streptococcus lutetiensis*  *Streptococcus oralis*  *Streptococcus salivarius*  *Corynebacterium striatum*  *Staphylococcus epidermidis*  *Cutibacterium namnetense*  *Streptococcus gordonii*  *Streptomyces cattleya*  *Kytococcus sedentarius*  *Enterococcus casseliflavus* | **28**  4  3  7  2  1  1  1  1  1  1  1  **─**  **─**  1  1  1  1  1 | **3**  **─**  **─**  **─**  **─**  1  **─**  **─**  **─**  **─**  **─**  **─**  1  1  **─**  **─**  **─**  **─**  **─** | **31**  4  3  7  2  2  1  1  1  1  1  1  1  1  1  1  1  1  1 |
| Anaerobic, No.  *Fusobacterium nucleatum*  *Prevotella melaninogenica*  *Bifidobacterium breve*  *Parabacteroides distasonis*  *Bacteroides fragilis*  *Clostridium innocuum*  *Bacteroides thetaiotaomicron*  *Clostridioides difficile*  *Bacteroides vulgatus*  *Prevotella oris*  *Veillonella dispar*  *Lactobacillus rhamnosus*  *Veillonella parvula*  *Lacticaseibacillus casei*  *Phocaeicola vulgatus*  *Porphyromonas gingivalis* | **17**  2  5  1  **─**  1  **─**  1  **─**  1  1  1  1  1  1  1  **─** | **6**  **─**  1  **─**  1  **─**  1  **─**  1  **─**  **─**  **─**  1  **─**  **─**  **─**  1 | **23**  2  6  1  1  1  1  1  1  1  1  1  2  1  1  1  1 |
| Viral, No.  CMV  EBV  HSV-1  Human adenovirus B  Human herpesvirus 7  Human adenovirus B  Torque teno virus  VZV  Kaposi sarcoma associated herpes virus | **30**  11  7  5  2  2  **─**  1  1  1 | **2**  1  **─**  **─**  **─**  **─**  1  **─**  **─**  **─** | **32**  12  7  5  2  2  1  1  1  1 |
| Fungi, No.  *Candida tropicalis*  *Candida albicans*  *Candida parapsilosis*  *Candida glabrata*  *Aspergillus flavus*  *Trichoderma atroviride* | **12**  5  2  1  2  1  1 | **2**  **─**  1  1  **─**  **─**  **─** | **14**  5  3  2  2  1  1 |
| Atypical, No.  *Mycobacterium abscessus*  *Mycoplasma hominis*  *Rickettsia typhi*  *Legionella longbeachae*  *Legionella pneumophila* | **4**  **─**  1  1  1  1 | **1**  1  **─**  **─**  **─**  **─** | **5**  1  1  1  1  1 |

**Supplemental Table 3. NGS versus Routine Tests Organism Identification**

|  |  | Fungal | Bacterial | Viral | Parasite | Total |
| --- | --- | --- | --- | --- | --- | --- |
| All Patients | NGS only | 8 | 65 | 19 | ─ | 92 |
|  | Routine only | 6 | 30 | 11 | 1 | 48 |
|  | Both | 6 | 22 | 13 | ─ | 41 |
| Adults | NGS only | 7 | 55 | 17 | ─ | 79 |
|  | Routine tests only | 5 | 18 | 2 | 1 | 26 |
|  | Positive for both | 4 | 15 | 13 | ─ | 32 |
| Pediatrics | NGS only | 1 | 10 | 2 | ─ | 13 |
|  | Routine tests only | 1 | 12 | 9 | ─ | 22 |
|  | Positive for both | 2 | 7 | ─ | ─ | 9 |

**Supplemental Table 4. Tests with Antimicrobials Changes Within 2 Days**

|  | Adults  (n=39) | Pediatric  (n=13) | Total  N=52 |
| --- | --- | --- | --- |
| NGS and Routine Tests Concordant, No. (%) | 4 (10) | 2 (15) | 6 (12) |
| NGS and Routine Tests Discordant, No. (%) | 11 (28) | 6 (46) | 17 (33) |
| NGS Positive and Routine Test Negative, No. (%) | 9 (23) | ─ | 9 (17) |
| NGS Negative and Routine Tests Positive, No. (%) | 3 (8) | 2 (15) | 5 (10) |
| NGS and Routine Tests Both Negative, No. (%) | 12 (31) | 3 (23) | 15 (29) |
| **Organisms Identified per Test, No. (%)**  0  1  2  3  4  5  6 | 15 (38)  11 (28)  8 (21)  **─** (0)  1 (3)  3 (8)  1 (3) | 5 (39)  6 (46)  2 (15)  ─ (0)  ─ (0)  ─ (0)  ─ (0) | 20 (39)  17 (33)  10 (19)  **─** (0)  1 (2)  3 (6)  1 (2) |

**Supplemental Table 5. Tests with Antimicrobials Changes Due to NGS**

|  | Adults  N=13 | Pediatrics  N=3 | Total  N=16 |
| --- | --- | --- | --- |
| NGS and Routine Tests Concordant, No. (%) | 1 (8) | 2 (67) | 3 (19) |
| NGS and Routine Tests Discordant, No. (%) | 7 (54) | 1 (33) | 8 (50) |
| NGS Positive and Routine Test Negative, No. (%) | 4 (31) | ─ (0) | 4 (25) |
| NGS Negative and Routine Tests Positive, No. (%) | ─ (0) | ─ (0) | ─ (0) |
| NGS and Routine Tests Both Negative, No. (%) | 1 (8) | ─ (0) | 1 (6) |
| **Organisms Identified per Test, No. (%)**  0  1  2  3  4  5  6 | 1  6 (46)  4 (31)  ─ (0)  1 (8)  ─ (0)  1 (8) | ─ (0)  3 (100)  ─ (0)  ─ (0)  ─ (0)  ─ (0)  ─ (0) | 1 (6)  9 (56)  4 (25)  ─ (0)  1 (6)  ─ (0)  1 (6) |

**Supplemental Table 6. NGS and Routine Tests Concordant**

|  | **Routine testing results** | **Date**  **Collected** | **NGS Result** | **Date Collected** | **Immuno-compromised** | **Change in therapy within 48 hours** |
| --- | --- | --- | --- | --- | --- | --- |
| 1 | Rickettsia Ab positive  EBV PCR blood positive | 3/15/23  3/15/23 | *Rickettsia typhi* EBV | 3/17/23 | Yes | No |
| 2 | Urine Antigen: *Streptococcus pneumoniae* | 11/26/22 | *Streptococcus pneumoniae* | 12/9/22 | No | No |
| 3 | Trach Aspirate: *Klebsiella aerogenes* | 9/14/22 | *Klebsiella aerogenes* | 9/10/22 | No | No |
| 4 | Lumbar Mass Tissue: *Propionibacterium (Cutibacterium)spp.* | 2/1/23 | *Cutibacterium namnetense* | 2/3/23 | No | No |
| 5 | Stool Culture: Vancomycin Resistant *Enterococcus* | 12/19/22 | *Enterococcus faecium* | 12/15/22 | Yes | Yes |
| 6 | Trach Aspirate: *Achromobacter spp. Stenotrophomonas maltophilia* | 4/15/23 | *Stenotrophomonas maltophilia Achromobacter ruhlandii* | 4/17/23 | No | No |
| 7 | Blood: CMV PCR positive | 12/23/22 | CMV | 12/30/22 | Yes | No |
| 8 | Blood: *Staphylococcus aureus*  Trach Aspirate: *Staphylococcus aureus* | 2/23/23  2/24/23 | *Staphylococcus aureus* | 2/25/23 | No | Yes |
| 9 | Blood: *Cardiobacterium* Species | 3/26/23 | *Cardiobacterium valvarum* | 3/29/23 | No | Yes* |
| 10 | Sputum: *Mycobacterium abscessus* | 11/06/22 | *Mycobacterium abscessus* | 11/10/22 | Yes | Yes* |
| 11 | Blood: EBV PCR positive | 11/2/22 | EBV | 11/1/22 | Yes | No |
| 12 | Blood: *Escherichia coli* | 10/10/22 | *Escherichia coli* | 10/14/22 | No | No |
| 13 | Blood: CMV PCR positive | 2/25/23 | CMV | 3/1/23 | No | No |
| 14 | Trach Aspirate: yeast | 4/20/23 | *Candida tropicalis* | 4/22/23 | No | Yes |
| 15 | BAL: yeast | 2/28/23 | *Candida glabrata* | 2/27/23 | Yes | Yes* |

Pediatric patients highlighted

*Antimicrobial change made due to NGS testing

**Supplemental Table 7. NGS and Routine Tests Discordant**

|  | **Routine testing results** | **Date resulted** | **NGS Result** | **Date resulted** | **Immuno-compromised** | **Change in therapy within 48 hours** |  |  |
| --- | --- | --- | --- | --- | --- | --- | --- | --- |
| 16 | BAL: *Pseudomonas aeruginosa* | 4/21/23 | *Pseudomonas aeruginosa* Human adenovirus B | 4/24/23 | No | No |  |  |
| 17 | Blood: *Candida parapsilosis* | 10/20/22 | *Candida parapsilosis Clostridium innocuum* | 10/26/22 | Yes | Yes |  |  |
| 18 | Trach Aspirate: *Pseudomonas aeruginosa* | 12/7/22 | *Pseudomonas aeruginosa Lactobacillus rhamnosus* | 12/5/22 | No | Yes |  |  |
| 19 | BAL: yeast | 5/16/23 | *Candida tropicalis Prevotella melaninogenica Prevotella oris Fusobacterium nucleatum Streptococcus gordonii* CMV HSV-1 | 5/17/23 | No | No |  |  |
| 20 | Blood: HHV-8 PCR positive  Stool Culture: Vancomycin Resistant Enterococcus | 2/11/23  2/10/23 | Kaposi sarcoma associated herpes virus HSV-1 *Enterococcus faecium* | 2/7/23 | Yes | No |  |  |
| 21 | Blood: CMV PCR positive  BAL: yeast | 1/26/23  1/20/23 | *Klebsiella quasipneumoniae* CMV *Enterococcus faecalis Candida tropicalis Human adenovirus B* | 1/23/23 | Yes | Yes |  |  |
| 22 | BAL: *Chryseobacterium spp*. | 3/30/23 | *Candida tropicalis* HSV-1 | 3/31/23 | No | Yes* |  |  |
| 23 | Urine: *Enterococcus spp.* | 2/25/23 | *Escherichia coli Enterococcus faecium Enterococcus faecalis Staphylococcus epidermidis Streptococcus lutetiensis* | 2/18/23 | Yes | Yes |  |  |
| 24 | Sputum: yeast | 2/23/23 | *Candida parapsilosis* Human herpesvirus 7 | 2/19/23 | Yes | Yes* |  |  |
| 25 | Urine: *Klebsiella pneumoniae* | 1/15/23 | *Streptococcus salivarius* | 1/16/23 | Yes | Yes* |  |  |
| 26 | Blood: CMV blood positive | 2/20/23 | CMV Candida tropicalis | 2/17/23 | No | Yes* |  |  |
| 27 | Synovial Fluid 16s: *Neisseria gonorrhoeae* | 11/18/22 | *Neisseria gonorrhoeae Candida glabrata* | 11/11/22 | No | Yes* |  |  |
| 28 | Sacral Wound: *Bacteroides thetaiotamicron*, *Enterococcus faecalis, Streptococcus anginosus* | 4/5/23 | *Bacteroides thetaiotamicron Bacteroides fragilis* | 4/12/23 | No | No |  |  |
| 29 | Blood: CMV PCR positive | 9/24/22 | CMV *Pseudomonas aeruginosa* | 9/20/22 | Yes | No |  |  |
| 30 | Trach Aspirate: *Enterobacter cloacae*  Blood: CMV PCR positive  Autopsy showed disseminated dematiaceous fungal abscesses, *Exserohilum ssp.* (fungal pericarditis, alveolar fungal abscess, GU abscess, thyroid fungal abscess) | 2/2/23  2/8/23  2/11/23 | *Enterobacter cloacae complex* CMV | 2/8/23 | No | No |  |  |
| 31 | Trach Aspirate: *Achromobacter spp., Stenotrophomonas maltophilia*  Wound Culture: *Stenotrophomonas maltophilia* | 5/22/23  5/23/23 | *Corynebacterium striatum* | 5/23/23 | No | Yes |  |  |
| 32 | BAL: *Staphylococcus aureus, Pseudomonas aeruginosa* | 4/4/23 | *Pseudomonas aeruginosa* | 4/6/23 | Yes | No |  |  |
| 33 | Blood: Adenovirus PCR positive    Nasal Swab: Parainfluenza 3 PCR positive | 6/22/22  6/25/22 | CMV *Candida albicans* | 6/24/22 | Yes | No |  |  |
| 34 | Brain Biopsy: toxoplasma by immunohistochemistry | 1/19/23 | CMV Human adenovirus B EBV  *Candida albicans* | 1/25/23 | Yes | No |  |  |
| 35 | Trach Aspirate: *Candida tropicalis* | 6/24/23 | *Pseudomonas aeruginosa* | 6/28/22 | Yes | Yes* |  |  |
| 36 | Trach Aspirate: *Stenotrophomonas maltophilia*  Blood: CMV PCR positive  Blood: VZV PCR positive | 4/25/23  4/30/23  5/1/23 | VZV CMV *Stenotrophomonas maltophilia Enterococcus faecium Staphylococcus epidermidis* | 4/26/23 | No | Yes |  |  |
| 37 | Sputum: *Stenotrophomonas maltophilia* | 2/15/23 | *Porphyromonas gingivalis* | 2/17/23 | No | Yes |  |  |
| 38 | CSF: CMV PCR positive | 10/22/22 | CMV EBV | 10/28/22 | No | No |  |  |
| 39 | Abdominal Abscess: *Acinetobacter spp., Enterococcus spp.*  Abdominal Abscess: *Mycobacterium intracellular chimera* | 4/13/23  4/20/23 | *Clostridioides difficile* | 4/13/23 | No | Yes |  |  |
| 40 | Dura Matter 16s: *Parageobacillus spp., Anoxybacillus spp.* | 11/15/22 | *Veillonella parvula* | 11/11/22 | Yes | No |  |  |
| 41 | Urine: *Serratia marcescens* | 2/15/23 | *Escherichia coli* | 2/22/23 | Yes | Yes |  |  |
| 42 | Blood: Parvovirus PCR positive | 3/11/23 | EBV | 3/7/23 | Yes | No |  |  |
| 43 | Blood: EBV PCR positive  Blood: *Candida glabrata* | 8/1/22  8/7/22 | EBV | 8/3/22 | Yes | Yes* |  |  |
| 44 | Urine: *Pseudomonas aeruginosa* | 3/1/23 | *Enterococcus raffinosus Pseudomonas aeruginosa Bifidobacterium breve Prevotella melaninogenica Lactobacillus rhamnosus Capnocytophaga sputigena* | 3/1/23 | Yes | Yes* |  |  |

Pediatric patients highlighted

*Antimicrobial change made due to NGS testing

**Supplemental Table 8. NGS Positive and Routine Tests Negative**

|  | **NGS Result** | **Date resulted** | **Immunocompromised** | **Change in therapy within 48 hours** |
| --- | --- | --- | --- | --- |
| 45 | *Staphylococcus epidermidis* | 5/9/23 | No | No |
| 46 | *Bacteroides vulgatus* | 10/25/22 | Yes | Yes* |
| 47 | *Pseudomonas aeruginosa* HSV-1 *Aspergillus flavus* | 10/12/22 | No | No |
| 48 | *Neisseria sicca Morococcus cerebrosus* | 2/14/23 | Yes | No |
| 49 | *Mycoplasma hominis* | 4/21/23 | Yes | No |
| 50 | *Pseudomonas aeruginosa* *Enterococcus faecalis* | 2/16/23 | No | Yes |
| 51 | *Prevotella melaninogenica* | 4/13/23 | Yes | No |
| 52 | *Escherichia coli* | 3/21/23 | No | No |
| 53 | *Prevotella melaninogenica* | 4/3/23 | Yes | Yes* |
| 54 | *Acinetobacter haemolyticus Trichoderma atroviride Prevotella melaninogenica Kytococcus sedentarius* | 1/5/23 | No | No |
| 55 | *Enterococcus faecalis* | 2/24/23 | Yes | No |
| 56 | *Prevotella melaninogenica* | 4/7/23 | No | No |
| 57 | *Staphylococcus epidermidis* | 2/13/23 | No | No |
| 58 | *Veillonella dispar Enterococcus faecalis* | 3/31/23 | No | Yes |
| 59 | *Pseudomonas aeruginosa* | 5/31/23 | Yes | No |
| 60 | *Enterococcus faecalis* CMV | 3/10/23 | No | Yes |
| 61 | HSV 7 | 2/4/23 | Yes | No |
| 62 | *Legionella pneumophila Rothia mucilaginosa* | 2/7/23 | Yes | Yes |
| 63 | *Fusobacterium nucleatum* | 7/28/22 | Yes | No |
| 64 | *Serratia ureilytica* | 2/27/23 | No | No |
| 65 | *Escherichia coli* | 4/19/23 | No | No |
| 66 | *Enterococcus casseliflavus* | 5/7/23 | No | Yes* |
| 67 | *Phocaeicola vulgatus* | 1/23/23 | Yes | Yes |
| 68 | *Parabacteroides distasonis* | 2/19/23 | No | No |
| 69 | *Streptococcus pneumoniae* *Pseudomonas pseudoalcaligenes* | 9/26/22 | No | No |
| 70 | *Rothia mucilaginosa Lacticaseibacillus casei Streptomyces cattleya Streptococcus oralis Rothia dentocariosa* | 4/13/23 | No | No |
| 71 | *Legionella longbeachae* HSV-1 EBV *Candida albicans* | 1/12/23 | Yes | Yes* |
| 72 | *Enterococcus faecalis* | 4/12/23 | No | No |
| 73 | Torque teno virus | 11/7/22 | Yes | No |

Pediatric patients highlighted

*Antimicrobial change made due to NGS testing

**Supplemental Table 9. NGS Negative and Routine Tests Positive**

|  | **Routine testing** | **Date resulted** | **NGS Date resulted** | **Immunocompromised** | **Change in therapy within 48 hours** |
| --- | --- | --- | --- | --- | --- |
| 74 | Blood: EBV PCR positive  Blood: HHV 6 PCR positive | 07/03/22 | 6/28/22 | Yes | No |
| 75 | Driveline Exit Wound: *Staphylococcus aureus* | 04/26/23 | 4/28/23 | No | No |
| 76 | Urine: *Klebsiella pneumonia* | 01/30/23 | 2/2/23 | Yes | No |
| 77 | 16S Bone: *Parageobacillus* spp*, Anoxybacillus* spp*., Streptococcus thermophilus* | 12/02/22 | 12/1/22 | Yes | No |
| 78 | Sputum: *Staphylococcus aureus* | 02/14/23 | 2/21/23 | No | No |
| 79 | Trach Aspirate: *Klebsiella pneumonia, Achromobacter* spp*., Stenotrophomonas maltophilia* | 01/30/23 | 1/3/23 | No | Yes |
| 80 | Neck Abscess: *Streptococcus parasanguinis, Veillonella* spp*, H. parainfluenzae* | 01/20/23 | 1/23/23 | No | No |
| 81 | SARS CoV2 PCR positive | 10/12/22 | 10/15/22 | No | No |
| 82 | BK virus blood PCR positive | 02/06/23 | 1/31/23 | Yes | No |
| 83 | Abdominal Wound: *Pseudomonas aeruginosa* | 02/02/23 | 2/8/23 | Yes | No |
| 84 | 28s on Sinus Tissue: *Rhizopus oryzae* | 1/13/23 | 2/20/23 | Yes | Yes |
| 85 | Trach Aspirate: *Staphylococcus aureus*, yeast | 05/21/23 | 5/22/23 | Yes | Yes |
| 86 | BAL: HSV PCR positive  Urine: *Candida albicans* | 05/26/23  05/18/23 | 5/23/23 | Yes | Yes |
| 87 | Pump Pocket Tissue: *Staphylococcus aureus* | 2/20/23 | 2/24/23 | Yes | No |
| 88 | Trach Aspirate: *Stenotrophomonas maltophilia* | 01/26/23 | 1/26/23 | No | Yes |
| 89 | Synovial Fluid: *Staph werneri, Staph hominis* | 12/19/22 | 12/15/22 | No | No |
| 90 | 16s Bone: *Prevotella intermedia* | 09/2/22 | 9/8/22 | No | No |

Pediatric patients highlighted

*Antimicrobial change made due to NGS testing
